# Supplementary material for: Ancient genomic variation underlies repeated ecological adaptation in young stickleback populations
Source: Evol Lett. 2018 Jan 26;2(1):9–21. doi: 10.1002/evl3.37 (PMC6121857; doi:10.1002/evl3.37)
Supplement: Supplementary file 1 — Figure S1. Longer sequences reduce variance in estimates of sequence diversity and divergence. Figure S2. Accurate phasing of RAD loci even at low population‐level sampling. Figure S3. RAD‐seq effectively samples genome‐wide sequence diversity. Figure S4. Relative (FST) and absolute (dXY) sequence divergence are positively correlated genome‐wide in two instances of marine‐freshwater divergence. Figure S5. TMRCA outlier regions remain outliers after removing highly differentiated RAD loci. Figure S6. Marine‐freshwater divergence in threespine sticklebacks is associated with reduced signal of monophyly of threespine haplotypes. Table S1. Sequencing summary for threespine stickleback samples. Table S2. Genome assembly statistics for Pungitius pungitius. [file EVL3-2-9-s001.pdf]

Supplementary figures and tables

Nelson, TC, and WA Cresko. **“Ancient genomic variation underlies repeated ecological adaptation in young stickleback populations”**

Figures:

S1. Longer sequences reduce variance in estimates of sequence diversity and divergence.

S2. Accurate phasing of RAD loci even at low population-level sampling.

S3. RAD-seq effectively samples genome-wide sequence diversity.

S4. Relative (F_{ST}) and absolute (d_{XY}) sequence divergence are positively correlated genome-wide in two instances of marine-freshwater divergence.

S5. T_{MRCA} outlier regions remain outliers after removing highly differentiated RAD loci.

S6. Marine-freshwater divergence in threespine sticklebacks is associated with reduced signal of monophyly of threespine haplotypes.

Tables:

Table 1. Sequencing summary for threespine stickleback samples

Table 2. Genome assembly statistics for *Pungitius pungitius*.

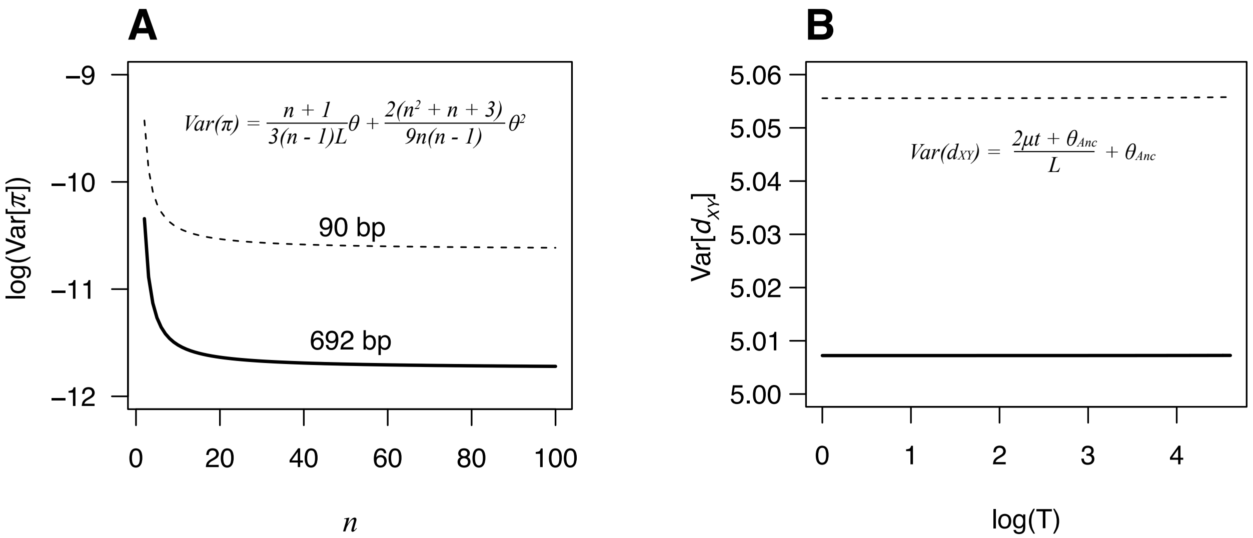

Figure S1. Longer sequences reduce variance in estimates of sequence diversity and divergence. A: Variance in π as a function of the number of chromosomes sampled, using sequence lengths typical of RAD-seq experiments (90 bp) and those in this study (692 bp). Variance was calculated using equation 10.9 in Nei (1987). Right: Variance in d_{XY} (using the equation in box 1 in Cruickshank and Hahn (2014)) as a function of (log-scaled) divergence time of two populations. The change in variance as function of divergence time is dwarfed by the difference in variance obtained with different sequence lengths. In both panels, n = sequences sampled; L = length of sequence sampled; $\theta = 4N\mu$; $\theta_{Anc} = \theta$ in the population ancestral to those sampled; μ = mutation rate per nucleotide; t = time since population split.

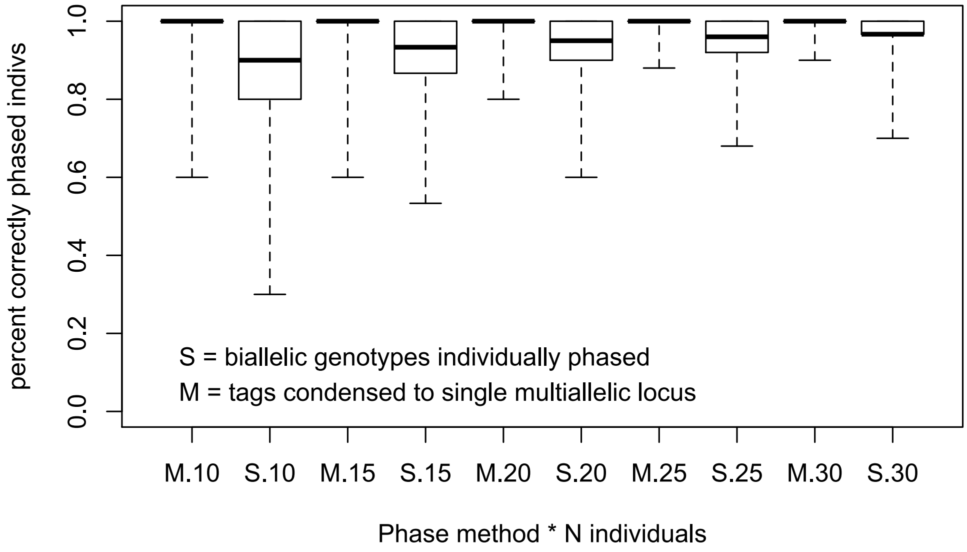

Figure S2. Accurate phasing of RAD loci even at low population-level sampling. Neutrally evolving, non-recombining RAD loci were simulated with ms and seq-gen to generate alignments with of 20 to 60 haplotypes (10-30 diploid individuals) and four to 30 segregating sites. Simulated haplotypes were then 'cut' at their midpoints and phased either by inputting all variable sites individually (biallelic 'SNPs', S) or by inputting the haplotype information on either site of the cut as multiallelic loci (M). Boxes represent interquartile range (IQR). Bold lines are medians. Whiskers extend to minimum and maximum values. Even with smaller sample sizes (10-15 individuals), over 75% of phasing attempts resulted in 100% phasing accuracy.

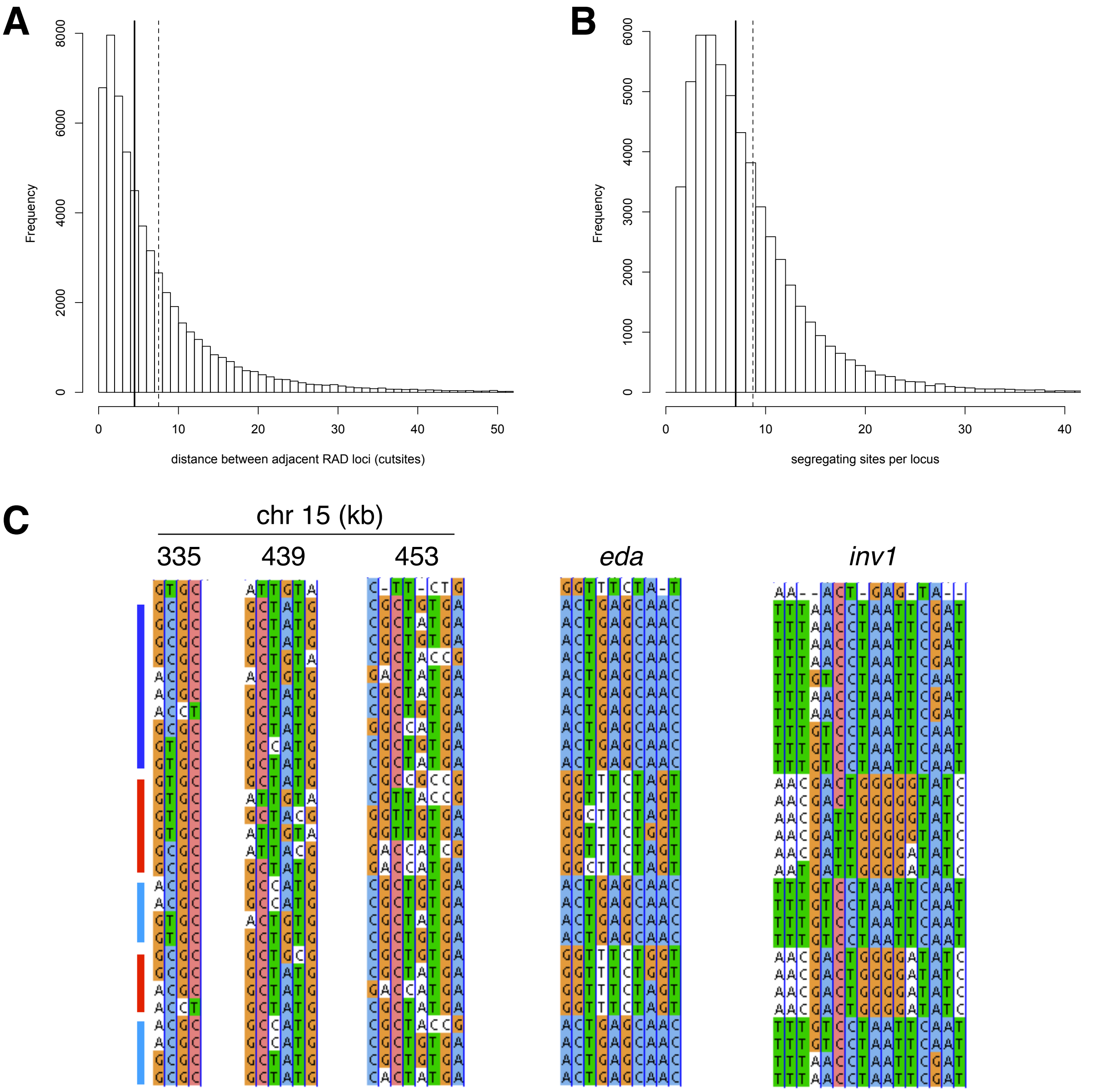

Figure S3. RAD-seq effectively samples genome-wide sequence diversity. Histograms of (A) the distance between adjacent RAD loci (calculated as the distance between the centers of each restriction site) and (B) the number of variable sites per locus show that most RAD loci were within 4 kb of their nearest neighbor and contained ≥ 7 variable sites. Means for each metric are shown as dashed vertical lines. Medians are solid lines. Each histogram is truncated to highlight the bulk of the distribution. Maximum values: distance = 455 kb; variable sites = 155. C: Example haplotypes from five RAD loci in non-divergent (chromosome 15) and divergent (*eda*, *inv1*) genomic regions. Chromosome 15 loci are labeled by their genomic position. The *eda* RAD locus is within the transcribed region of *eda* and *inv1* is within the breakpoints of the chromosome 1 inversion. Colored bars identify population/ecotype of origin. Red: RS (marine); dark blue: BL (freshwater); light blue: BP (freshwater). Alignments visualized in JalView v1.0 (Waterhouse, *et al*, 2009). Only sites that are variable within threespine stickleback are shown.

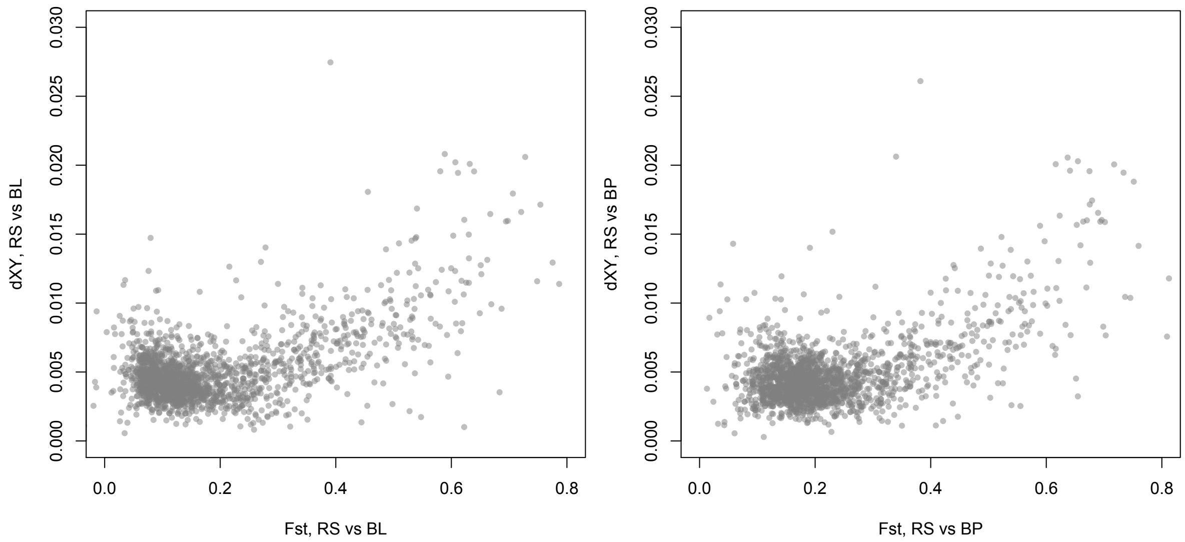

Figure S4. Relative (F_{ST}) and absolute (d_{XY}) sequence divergence are positively correlated genome-wide in two instances of marine-freshwater divergence. Points are 250 kb non-overlapping genomic windows. Left panel compares the marine Rabbit Slough population (RS) to the freshwater Boot Lake population (BL) (type-II linear model: $r^2 = 0.314$, permuted p-value [reduced major axis] = 0.01). Right panel compares RS to the freshwater Bear Paw Lake population (BL) (type-II linear model: $r^2 = 0.311$, permuted p-value [reduced major axis] = 0.01).

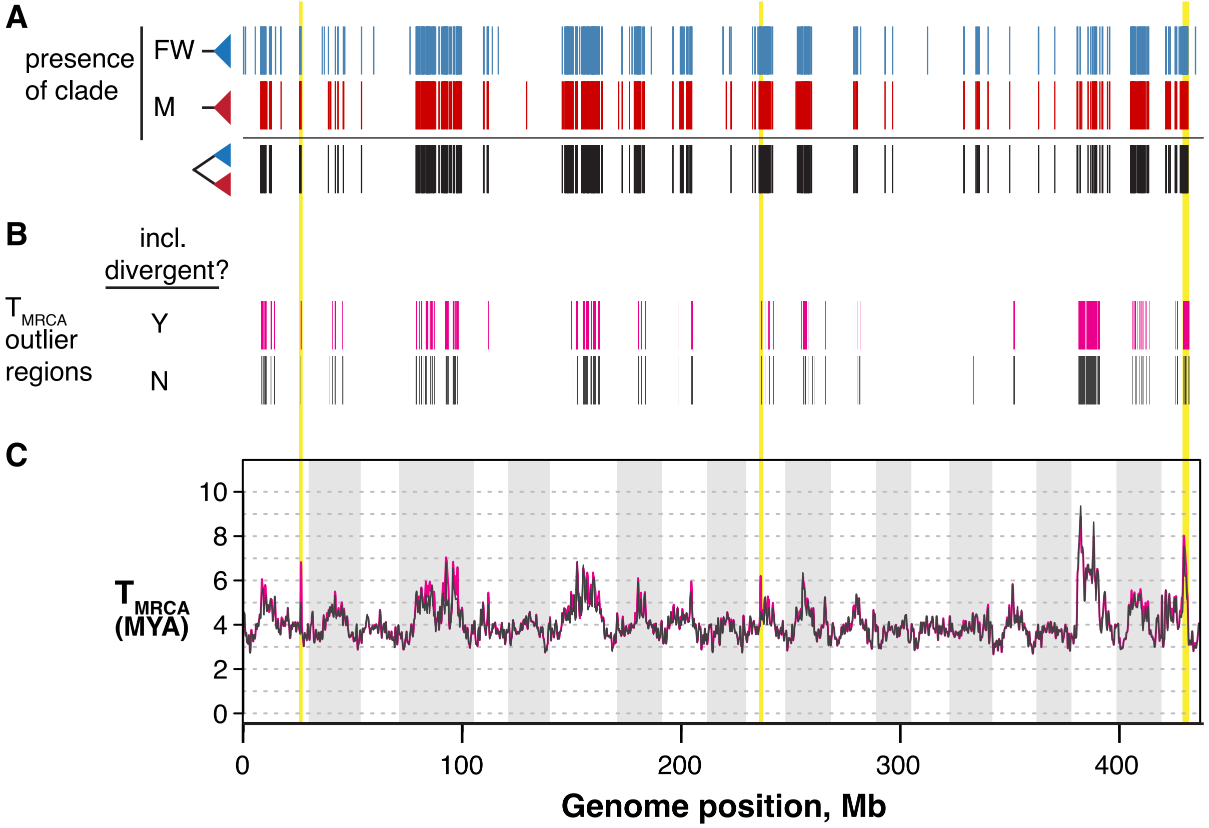

Figure S5. T_{MRCA} outlier regions remain outliers after removing highly differentiated RAD loci. Panel A is taken from Fig. 2 and shows the genomic distribution of reciprocally monophyletic ("divergent"; black bars) RAD loci. Panel B shows the distributions of T_{MRCA} outlier regions (increased T_{MRCA}) including all RAD loci (magenta boxes, "Y"). Below are the T_{MRCA} outlier regions after removing divergent loci and any RAD locus with a marine-freshwater (RS vs. [BL+BP]) $F_{ST} > 0.5$, which is approximately the top 7% of the F_{ST} distribution. Panel C: Genome scans of T_{MRCA} using all RAD loci (magenta) and excluding marine-freshwater outliers (gray).

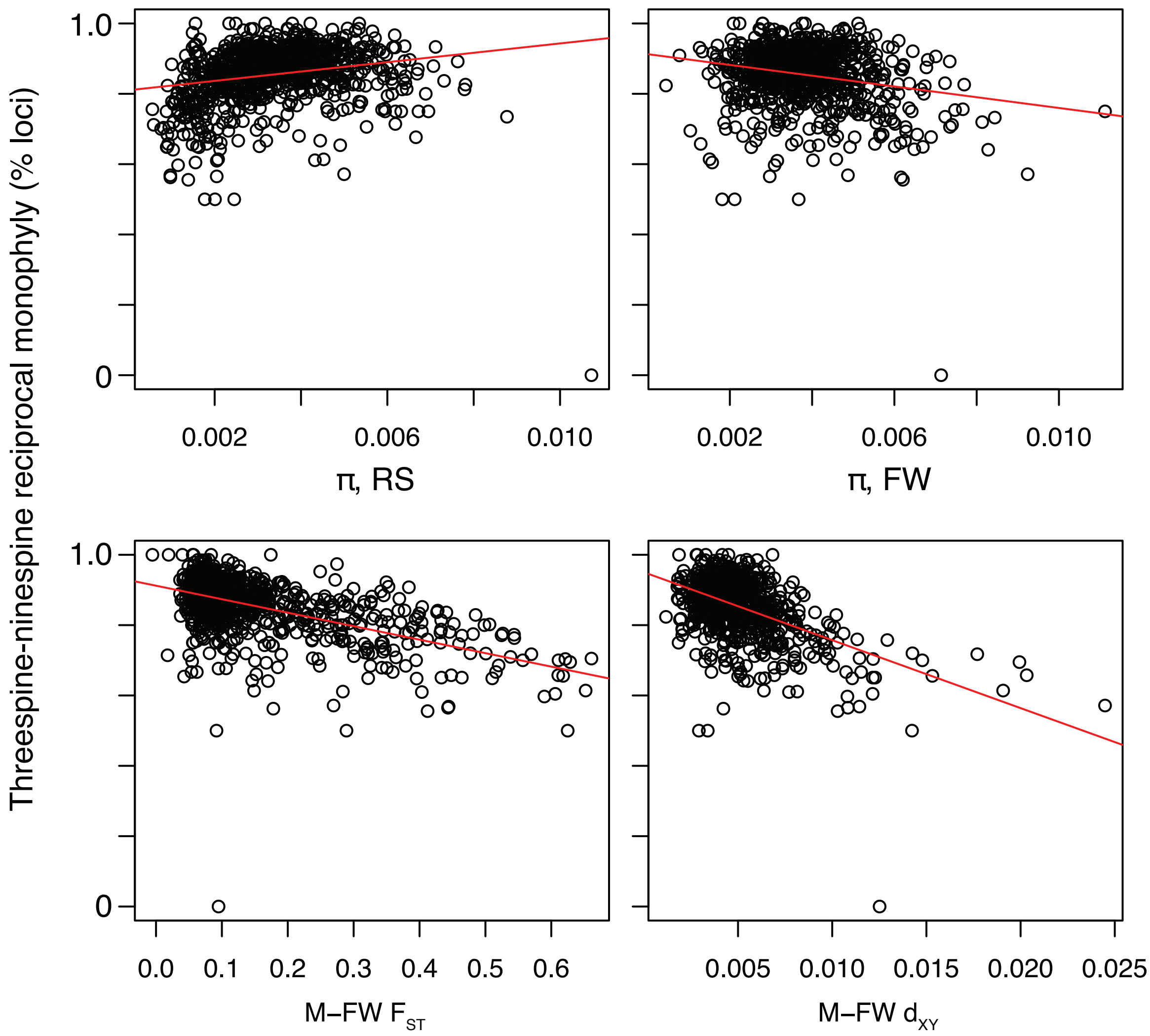

Figure S6. Marine-freshwater divergence in threespine sticklebacks is associated with reduced signal of monophyly of threespine haplotypes. All y-axes give the fraction RAD loci in a given genomic window in which threespine stickleback haplotypes were monophyletic to the exclusion of the ninespine haplotype. Points represent non-overlapping 500-kb genomic windows.

Supplementary table 1. Sequencing summary for threespine stickleback samples

**Sample**	population	raw reads	filtered reads	merged pairs	mean coverage per locus
1827.05	Rabbit Slough	10167407	10031967	7269377	12X
1827.06	Rabbit Slough	10265078	10172621	7591801	13X
1827.07	Rabbit Slough	9175983	9040625	6771332	11X
1827.08	Rabbit Slough	7896938	7814081	5879351	10X
1827.09	Rabbit Slough	8773502	8668261	6405777	11X
2827.01	Boot Lake	8917575	8810382	6373001	11X
2827.07	Boot Lake	10064876	9917732	7255989	13X
2827.13	Boot Lake	9099831	9002717	6528704	12X
2827.19	Boot Lake	11021084	10792092	7911026	14X
2827.25	Boot Lake	9920574	9814758	7287485	13X
1902.02	Bear Paw Lake	4780489	4365505	2942926	5X
1902.03	Bear Paw Lake	5073434	4643909	3192582	5X
1902.04	Bear Paw Lake	4902931	4600877	3138791	6X
1902.06	Bear Paw Lake	4501906	4339253	2983345	5X

Supplementary table 2. Genome assembly statistics for *Pungitius pungitius*.

	contig (scaffold)
n	393,037 (391,396)
Max length (bp)	165,088 (182,644)
N50 (bp)	9,202 (9,886)
Average length (bp)	1,314 (1,320)
Gaps (%)	0.03
Total assembly length (bp)	516,674,741
